# Humpback Whale Song on the Southern Ocean Feeding Grounds: Implications for Cultural Transmission

**DOI:** 10.1371/journal.pone.0079422

**Published:** 2013-11-20

**Authors:** Ellen C. Garland, Jason Gedamke, Melinda L. Rekdahl, Michael J. Noad, Claire Garrigue, Nick Gales

**Affiliations:** 1 National Marine Mammal Laboratory, Alaska Fisheries Science Center, National Marine Fisheries Service, National Oceanic and Atmospheric Administration, Seattle, Washington, United States of America; 2 Ocean Acoustics Program, Office of Science and Technology, National Marine Fisheries Service, National Oceanic and Atmospheric Administration, Silver Spring, Maryland, United States of America; 3 Cetacean Ecology and Acoustics Lab, School of Veterinary Science, University of Queensland, Gatton, Queensland, Australia; 4 Opération Cétacés, Noumea, New Caledonia; 5 Australian Marine Mammal Centre, Australian Antarctic Division, Kingston, Tasmania, Australia; University of St Andrews, United Kingdom

## Abstract

Male humpback whales produce a long, complex, and stereotyped song on low-latitude breeding grounds; they also sing while migrating to and from these locations, and occasionally in high-latitude summer feeding areas. All males in a population sing the current version of the constantly evolving display and, within an ocean basin, populations sing similar songs; however, this sharing can be complex. In the western and central South Pacific region there is repeated cultural transmission of song types from eastern Australia to other populations eastward. Song sharing is hypothesized to occur through several possible mechanisms. Here, we present the first example of feeding ground song from the Southern Ocean Antarctic Area V and compare it to song from the two closest breeding populations. The early 2010 song contained at least four distinct themes; these matched four themes from the eastern Australian 2009 song, and the same four themes from the New Caledonian 2010 song recorded later in the year. This provides evidence for at least one of the hypothesized mechanisms of song transmission between these two populations, singing while on shared summer feeding grounds. In addition, the feeding grounds may provide a point of acoustic contact to allow the rapid horizontal cultural transmission of song within the western and central South Pacific region and the wider Southern Ocean.

## Introduction

Male humpback whales produce a long, stereotyped and constantly evolving vocal breeding display, termed ‘song’ [1], [2]. Within a population, males conform to the current arrangement and content of the song [3]–[5]. The conformity to a single song type within a population is thought to occur via vocal learning from surrounding males and, when song transmission is examined at the ocean basin scale, is considered one of the best examples of horizontal cultural transmission in a non-human animal [6]. Song similarity has also been documented between populations, although the degree to which this occurs is dependent upon the geographical distance between such populations [6]–[13]. Thus, song similarity among populations indicates that acoustic contact is likely to have occurred, although there is currently little known about the mechanism(s) through which song transmission is mediated. Therefore, identifying all potential mechanisms of transfer is essential to understanding the dynamics of song transmission within and across regions.

Within an ocean basin, populations in closer proximity to each other typically display a higher degree of song similarity [6]–[12]. In the North Pacific, for example, studies of song sharing have shown that the geographically close populations of Hawaii and Mexico shared a higher number of themes, compared to Japan [8]–[9], [11]. Similarly, in the Southern Hemisphere, songs recorded across the South Atlantic were similar across the ocean basin within a year [12]. In more geographically isolated populations, such as in the Indian Ocean (Madagascar and western Australia), little song sharing was found to occur across the ocean basin (shown through a single shared theme [14]).

Typically, humpback whales from different ocean basins sing distinctly different songs (see [3]–[4], [7], [15]–[16]). Despite this, song from Gabon (South Atlantic) was found to be similar in a single year (2003) to song from Madagascar (Indian Ocean) [17]. Similarly, song from the western Australian population (Indian Ocean) has spread into the eastern Australian population (South Pacific) [13], although little is known about how songs are shared between these two populations.

Payne and Guinee [7] hypothesized three possible mechanisms to allow acoustic contact and subsequent song transmission among populations in any ocean basin. The first possibility is movement of individuals from one breeding population to another between seasons (a phenomenon which has been observed repeatedly; see [18]–[21]). The second is within-season movement of individuals between two breeding populations (rarely observed; see [19]–[20]). The third is song exchange on shared migration routes and/or on summer feeding grounds in high latitudes.

Although high-latitude song is, compared to that on breeding grounds, relatively uncommon, such singing has been observed in spring, summer or autumn in the North Atlantic [22]–[24], the North Pacific [25], and off the Western Antarctic Peninsula [26]. It has also been recorded extensively during migration, including off the eastern coast of Australia [16], [27], in the North Pacific [28] and the North Atlantic [1], [29]–[30], and off New Zealand [10], [31]. Song transmission among populations within the western and central South Pacific region is more likely to occur through the movement of individual males between seasons and/or singing while on shared migratory routes, than by males moving between populations in a single season [6], [13], as these movements, although documented, are rare in comparison to both inter-seasonal movements of males or migratory song [20]. Although mixing of populations on the feeding grounds has been reported (between eastern and western Australian whales [32]), singing while on these shared summer feeding grounds has not been reported for the South Pacific populations, or the greater Southern Ocean away from the Western Antarctic Peninsula.

**Figure 1:**
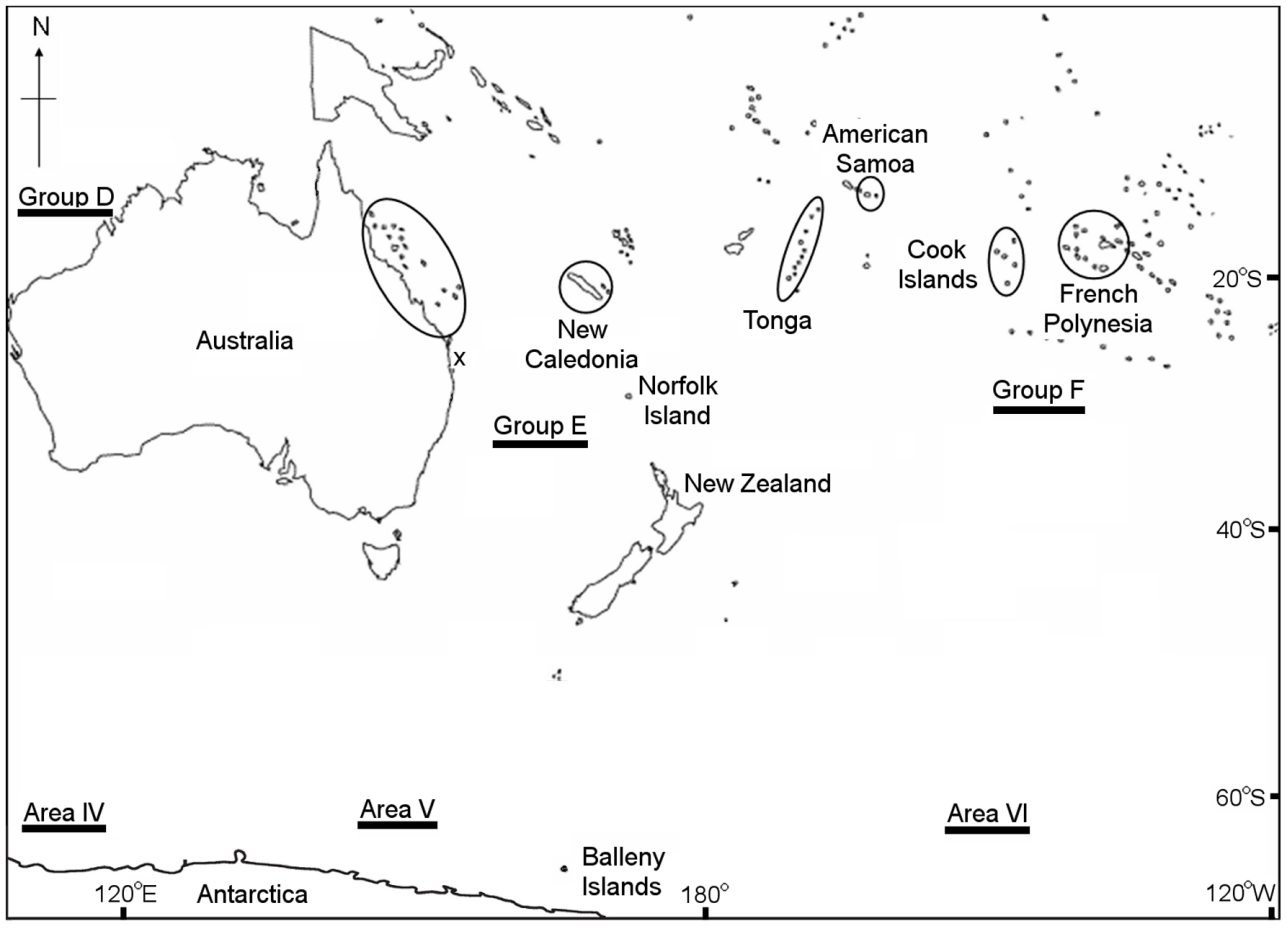
Map of the South Pacific and corresponding Antarctic region. Suggested breeding groups (D, E, and F) and corresponding Antarctic feeding areas (IV, V and VI) are shown. Circles represent the location of the major breeding populations discussed within the region (see text). Recordings in eastern Australia were taken at Peregian Beach, SE Queensland, while animals where on migration (x).

A number of humpback whale populations have been recognised around the world due to strong feeding and/or breeding ground site fidelity [18], [33]–[35]. In the western and central South Pacific region, the International Whaling Commission (IWC) currently recognizes two different breeding stocks (E and F [36]). These are thought to migrate to two corresponding feeding regions in Antarctica (Areas V, 120°E to 180° W, and VI, 180° W to 120° W [36]). Group E is further divided into ‘sub-stocks’ E1 eastern Australia, E2 New Caledonia and E3 Tonga, while, further to the east, Group F is divided into F1 Cook Islands and F2 French Polynesia [36] (Figure 1). Recovery from commercial whaling, including the severe overexploitation from illegal Soviet whaling [37], has been uneven within the region. The eastern Australian population is recovering strongly with a high rate of population growth [38]–[39], while other populations within the Oceania region are showing little signs of recovery [40]–[41].

Linkages between breeding and feeding grounds have been demonstrated in the South Pacific using ‘Discovery marks’, small metal cylinders shot into whales during whaling and subsequently recovered when whales were later killed [32], [42]–[44]. These provided linkages in the 1950s and early 1960s between Antarctic feeding Area V and Tonga, Norfolk Island, New Zealand [43], and eastern Australia [32]. More recently, genetic analyses (*e.g.,* [45]), photo-identification comparisons (*e.g.,* [46]) and satellite tagging studies (*e.g.,* [47]) have demonstrated connections between feeding and breeding areas. The dedicated Australia-New Zealand Antarctic Whale Expedition to Antarctic Area V in 2010 [48] and project CETA in 2010 [49], highlighted connections between the Balleny Islands region (66–68°S and 162–165°E) and waters off Adelie Land (65–66°S and 140–145°E) and the eastern Australian migratory corridor (six genetic matches, 23 photo-identification recaptures for the Balleny Islands, one photo-identification recapture for Adelie Land), the New Zealand migratory corridor (one genetic match to the Balleny Islands), and the New Caledonian breeding ground (one photo-identification recapture for the Balleny Islands) [45]–[46]. Opportunistic acoustic recordings were taken as part of the Australia-New Zealand Antarctic Whale Expedition and are analysed here to assess the similarity between humpback whale song recorded in the Southern Ocean and other regions.

Song sharing within the western and central South Pacific is very dynamic (based on a multi-year, multi-population song analysis) [6], [50]–[51]: songs have been documented radiating repeatedly across the region from west to east, from eastern Australia to French Polynesia, usually over a period of two years. The inclusion of song from the feeding grounds into such analyses represents a significant opportunity to acoustically link breeding to feeding grounds, and to explore how mechanisms of song transmission may contribute to the large-scale pattern of song similarity across this vast oceanographic region.

Here we report the first recorded occurrence of humpback whale song in Antarctic Area V, south of the eastern Australian and New Caledonian breeding populations and their shared migratory corridor, New Zealand. Although song was available from only faint singers, we were able to identify the song as being humpback whale in its origin, and then to compare the song from both eastern Australia and New Caledonia in the previous and following breeding seasons, in an attempt to acoustically link breeding to feeding grounds.

## Materials and Methods

During the dedicated Australia-New Zealand Antarctic Whale Expedition on *RV Tangaroa* [48], sonobuoy recordings containing presumed humpback whale song were made from sonobuoys deployed in a region 150–200 km southwest of the Balleny Islands, and 90–150 km north of the Antarctic continent. These occurred over a 48 hour period (March 5–7, 2010; Table 1) and spanned roughly a 70 km range, with the southernmost sonobuoy deployed at 69° 18′ S, 166° 16′ E and the northernmost sonobuoy deployed at 68° 43′ S, 166° 53′ E. Recordings were made of underwater sound transmitted from radio-linked sonobuoys (DIFAR 53D, functional audio range of 10 Hz to at least 2.4 kHz; see [52] for further details). Sound was digitized (wav file format, 12 bit, 48 kHz sampling rate) on a National Instruments PCMCIA DAQCard-6062E and recorded onto a laptop running *Ishmael* software [53]. A total of one hour of sound files (∼5 minute wav files downsampled to 6 kHz sampling rate) with identifiable song were examined (Table 1). Songs from eastern Australia were recorded at Peregian Beach, Queensland, using moored, radio-linked hydrophone buoys (a brief description is provided below; see [27], [54] for additional details on equipment set up). These had High Tech HTI 96 MIN hydrophones with a built-in +40 dB gain pre-amplifier and an additional external custom-built preamplifier (+20 dB). The signals were transmitted using AN/SSQ-47A sonobuoy transmitters and received onshore using a type 8101 sonobuoy receiver. The radio signals were recorded directly to computer (wav file format, 16 bit, 22 kHz sampling rate) running *Ishmael* software [53] using a National Instruments E-series data acquisition card. Songs from the southern lagoon of New Caledonia were recorded using a single High Tech HTI 96 MIN hydrophone with built-in +40 dB gain pre-amplifier and M-Audio Microtrack 24/96 digital recorder (wav file format, 16 bit, 44.1 kHz sampling rate). Ethical and permit approval for this work was obtained from all appropriate organizations (The University of Queensland Animal Ethics Committee, The Australian Federal Government, The Queensland State Government and Direction de l’Environnement Province Sud, New Caledonia).

**Table 1:** Summary of recordings that contained suspected humpback whale song in Antarctic Area V. Bold indicates the highest quality recordings. See Figure 2 for corresponding spectrograms.

Date	Time	Length(hr:min:sec)	Humpbackvocalizations present	Clear songpattern (Y/N)	Themes identified
5-Mar-10	19∶47∶33	5∶00	Y	N	
	19∶52∶33	5∶00	Y	Y	Theme H
	19∶57∶33	5∶00	Y	N	
	20∶02∶33	5∶00	Y	N	
6-Mar-10	18∶45∶00	5∶00	Y	N	
	**19∶45∶41** *	4∶35	Y	Y	**Theme I, K & M1**
	**19∶53∶02** *	3∶40	Y	Y	**Theme H, I & J**
	**19∶58∶50**	2∶00	Y	Y	**Theme H & K**
	20∶20∶00	5∶00	Y	Y	Theme K
7-Mar-10	06∶46∶48	5∶00	Y	Y	Theme K
	06∶51∶48	5∶00	Y	N	
	08∶26∶48	5∶00	Y	Y	Theme M1
	08∶31∶48	5∶00	Y	N	
	08∶36∶48	5∶00	Y	Y	Theme M1
	17∶47∶05	5∶00	Y	Y	Theme I & M1
	17∶52∶05	5∶00	Y	Y	Theme H & I
	**17∶57∶05**	5∶00	Y	Y	**Theme H & I**
	18∶02∶05	5∶00	Y	N	
	18∶22∶05	5∶00	Y	Y	Theme K
	18∶27∶05	5∶00	Y	N	
	19∶55∶30	5∶00	Y	Y	Theme M1
Total time examined		1∶40∶15			
Time with song		1∶00∶15			

**Figure 2:**
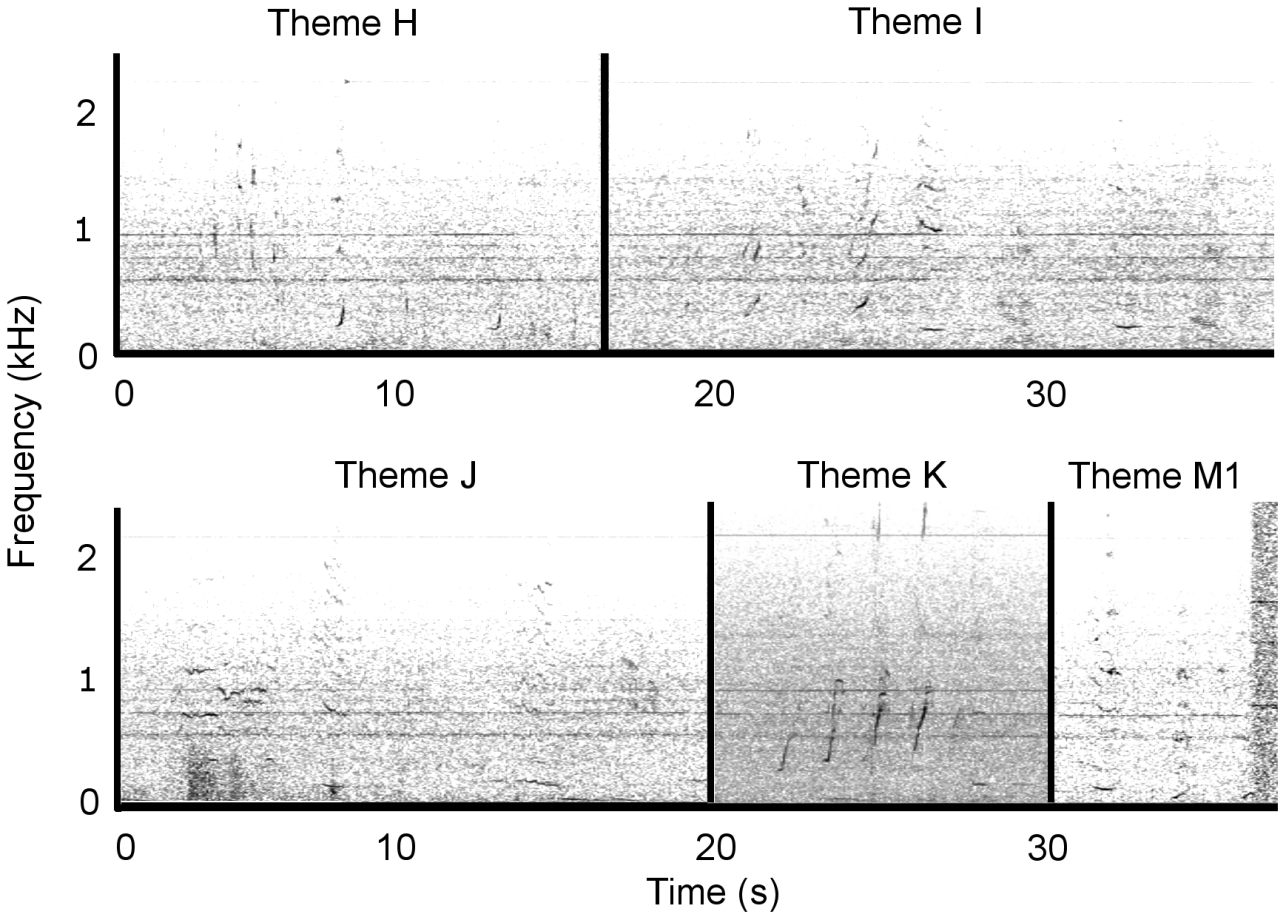
Antarctic Area V 2010 song. A single representative phrase is shown for each theme representing a collection of separately identified themes. While the themes illustrated were taken from an almost continuous ten minute recording session (see Table 1), no clear theme sequence was discernible. Spectrograms were generated in Raven Pro 1.4 (Blackman-Harris window, 1024 FFT size, 75% overlap). Recordings were taken from 5 to 7 March 2010. Audio is provided in acoustic file S1. Note the difference in frequency scale for Figure 2 compared to Figures 3 to 6.

* Spectrograms in Figure 2 were taken from these recordings.

Songs were viewed in Abode Audition (2.0 and CS5.5) using Blackman-Harris, 75% overlap, 2048 point fast Fourier transform (FFT) for the eastern Australian and New Caledonian recordings and 1024 point FFT for the Antarctic Area V recordings (to ensure a comparable frequency resolution), displaying approximately 30 seconds of song from 0–2.5 kHz. The nature of the Antarctic recording permitted only qualitative analysis of the song. First, to identify if the sounds were humpback whale song, all sound units were examined to see if there were any repeating patterns. Each sound type (‘unit’) was assigned a descriptive name based on the visual and aural qualities of the sound (*e.g.,* ‘ascending cry’, ‘moan’, ‘purr’; see [6], [55] for unit descriptions and qualitative unit identification). Humpback whale song is composed of multiple sounds (‘units’) that make a stereotyped pattern (a ‘phrase’); these are then repeated multiple times to make a ‘theme’ [1]. A few themes, which are sung in a particular order, comprise a song. Identifying repeated, stereotyped phrases within the vocalization strongly suggests the sound is produced by a male humpback whale and constitutes a ‘song’. Multiple potential instances of song were present in some of the Antarctic recording as humpback whale vocalizations/units were recorded; these were not included in further analysis unless a clear phrase pattern was present indicating song (Table 1). Second, once phrases were identified for the Antarctic sample, 2009 and 2010 song from eastern Australia and New Caledonia were examined (Table 2). This was done by comparing the themes for each year and location. For the eastern Australia 2009 and 2010 samples, previous examination of the songs identified all themes that were present within each year of song (M. L. Rekdahl, E. C. Garland and A. Murray unpublished data). For the New Caledonian song for both years, high quality recordings were examined in Adobe Audition.

**Table 2:** Sample sizes of the number of singers and the total number of songs for eastern Australia and New Caledonia in 2009 and 2010.

Location	Year	Number of singers	Total number of songs
Eastern Australia	2009	6	32
	2010	6	30
New Caledonia	2009	3	14
	2010	6*	14

* One singer sung the New Caledonian 2009 song type.

For each location and year, all themes were described by summarising the units used by each singer for every phrase. In some themes there were alternate forms of phrases where one sound type was replaced by another. To ensure theme classification and thus matching was as objective as possible and repeatable, three naïve observers (a bowhead whale song specialist, a killer whale and right whale call specialist, and a fin whale call specialist with humpback whale song experience) were asked to match a number of themes. Ten themes in total (a reference set) were chosen from the song types presented in the current study. These were displayed (along with the test set) in Raven Pro 1.4 (using the same settings as for the analysis) to allow the observers to both visually and aurally assess each theme. Each observer was given the test set containing 20 themes and was asked to assign each sample with a theme number or, in the case of one sample, no matching theme. The observers classified 89%, 90% and 95% of themes in a similar manner, resulting in an average 91% agreement in classification. Mismatches were between themes M1 (eastern Australia 2010) and M2 (eastern Australia 2010), and through the evolution of the eastern Australian 2010 theme H to include ‘whoops’ in the squeak series. All matches pertaining to the 2009 eastern Australian, New Caledonian 2010 and Antarctic Area V themes resulted in 100% agreement in classification. Humpback whale song themes are known to be highly variable with a large number of sound types that can be combined in many different ways. For example, Oceania song over the period 1998 to 2008 has had at least 93 phrase types [55]. Therefore, the chance of two themes matching by chance is extremely small.

## Results

**Figure 3:**
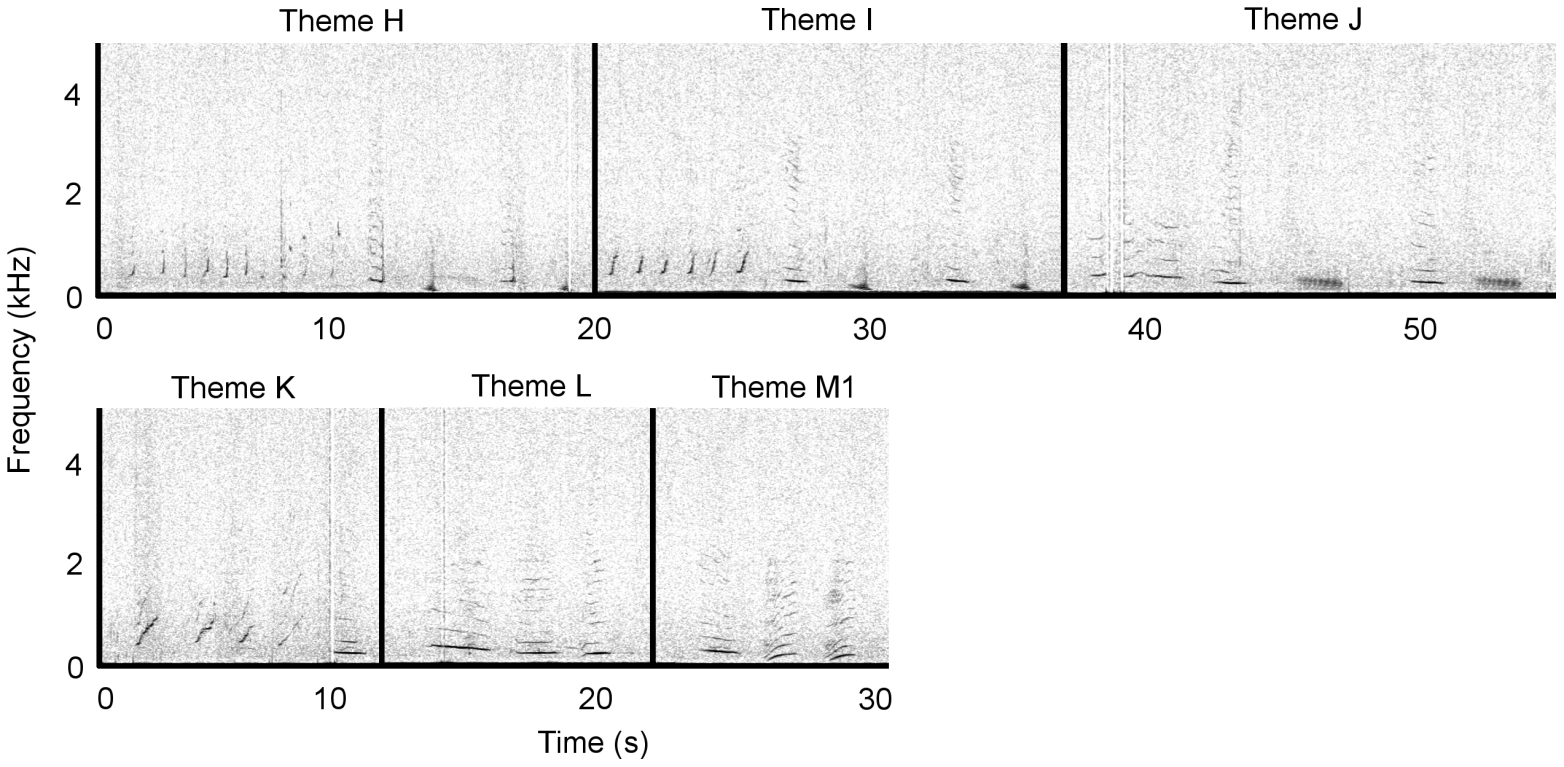
Eastern Australia 2009 song. A single representative phrase is shown for each theme representing a song. Spectrograms were generated in Raven Pro 1.4 (Blackman-Harris window, 2048 FFT size, 75% overlap). Recordings were taken in October 2009. Audio is provided in acoustic file S2. Note difference in frequency scale to Figure 2.

**Figure 4:**
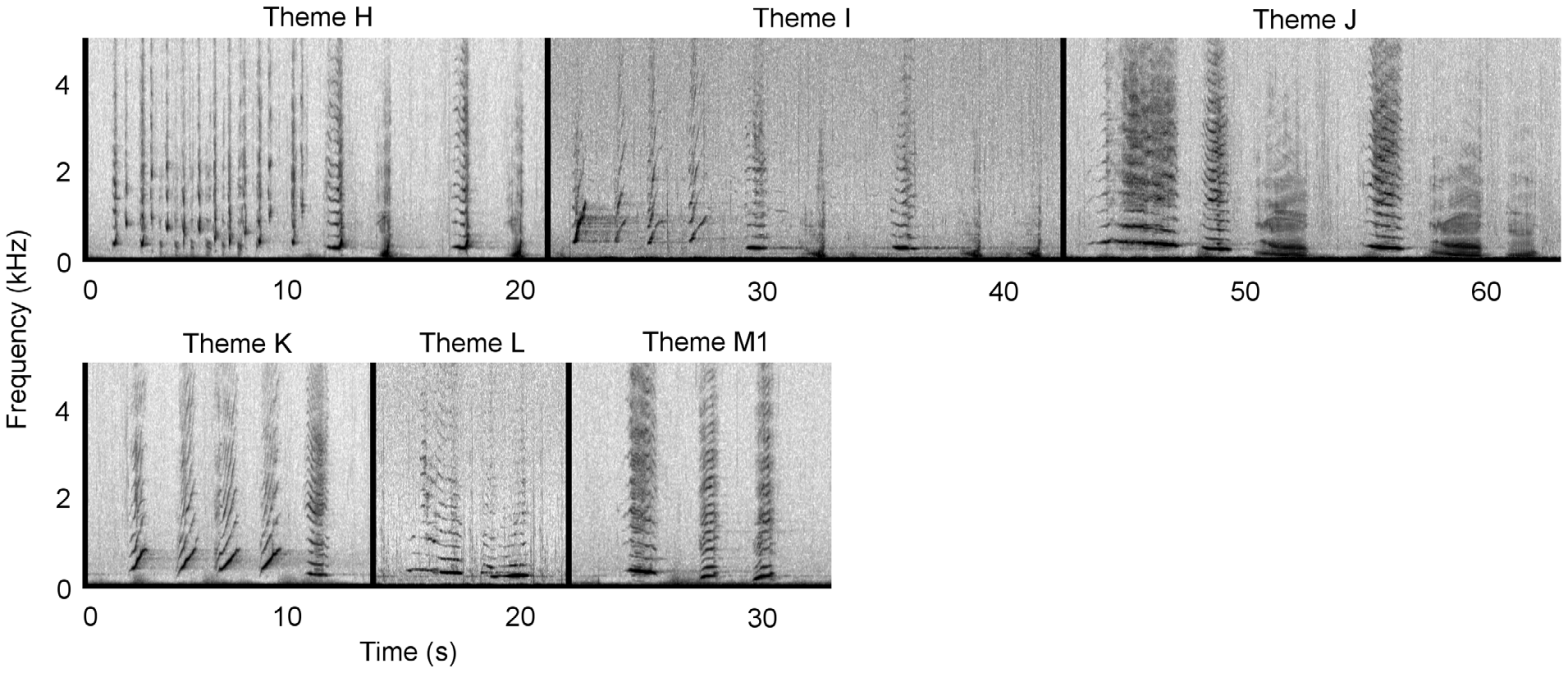
New Caledonia 2010 song. A single representative phrase is shown for each theme representing a song. Spectrograms were generated in Raven Pro 1.4 (Blackman-Harris window, 2048 FFT size, 75% overlap). Recordings were taken from July to September 2010. Audio is provided in acoustic file S3. Note difference in frequency scale to Figure 2.

Due to the repetition of a stereotyped sequence of species-typical sounds, the recorded sounds from Antarctic Area V were identified as humpback whale song. At least four stereotyped phrases and thus themes (labelled H to M) were identified (Figure 2); these qualitatively matched four themes from the eastern Australian 2009 song (Figure 3) and the same four themes from the 2010 New Caledonian song (Figure 4).

### Song Description for Eastern Australia 2009 and 2010, New Caledonia 2010 and Antarctic Area V 2010 Phrases

**Figure 5:**
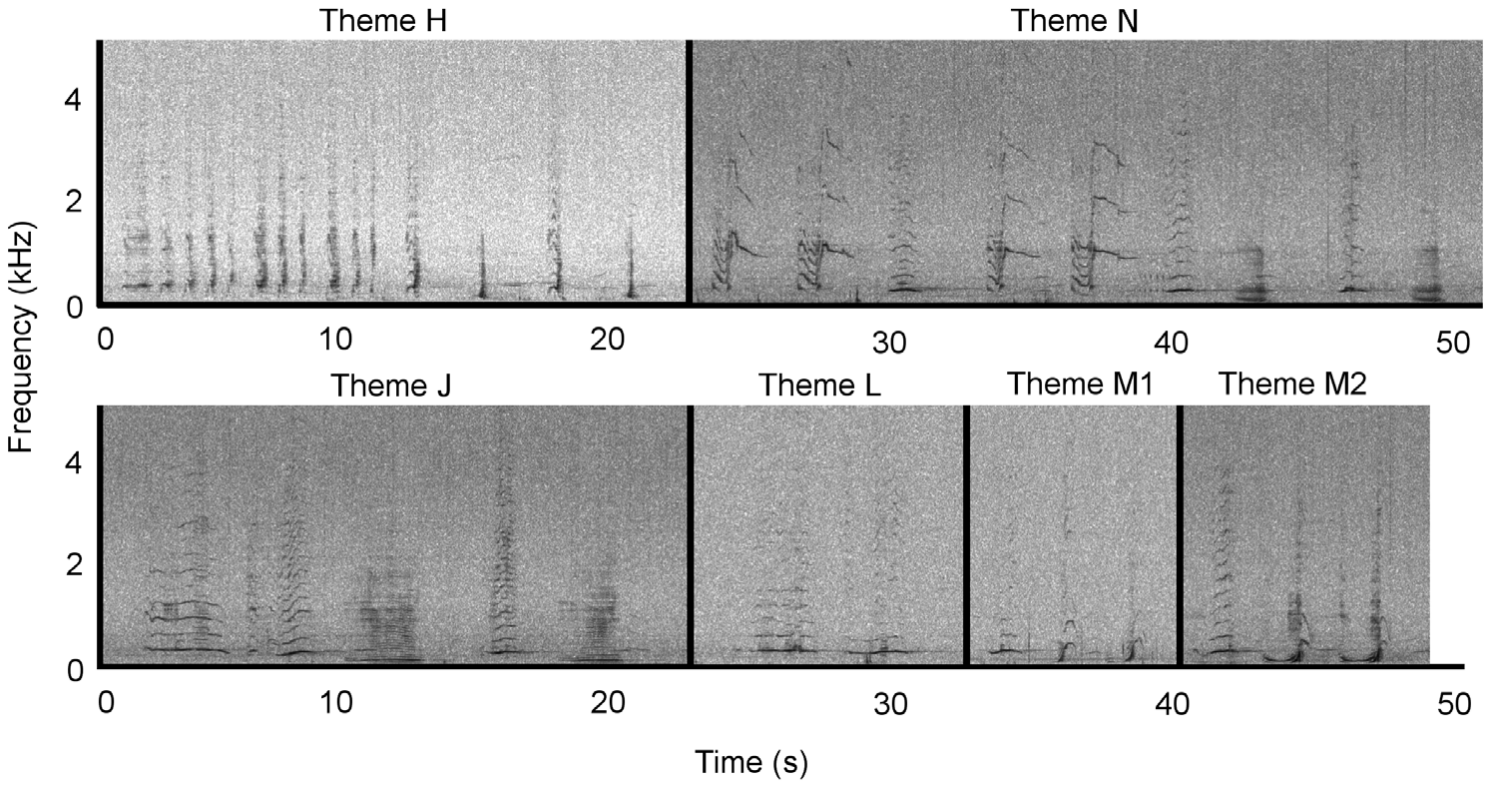
Eastern Australia 2010 song. A single representative phrase is shown for each theme representing a song. Some themes contained 1 and 2 phrase types representing small but stereotyped variations to a theme. Spectrograms were generated in Raven Pro 1.4 (Blackman-Harris window, 2048 FFT size, 75% overlap). Recordings were taken from September to October 2010. Audio is provided in acoustic file S4. Note difference in frequency scale to Figure 2.

Four themes from the Antarctic Area V sample were seen repeatedly (H, I, K & M1; Table 1), but no sequence of themes was discernible (Figure 2, Acoustic file S1). Theme J was heard on a single occasion in the Antarctic Area V sample, and as such is only considered a potential match. Six themes were present in the eastern Australian song in 2009 (Figure 3, Acoustic file S2). Themes were typically sung H, I, J, K, L and M1. In 2010, theme I evolved into theme N, and theme M1 progressed into a second phrase, M2. Thus, the theme sequence was H, N, J, L, M1 and M2 (Figure 5, Acoustic file S4). The New Caledonian 2010 song sequence predominantly consisted of themes H, I, J, K, L and M1 (note description below of an individual singing the 2009 song in New Caledonia in 2010; Figure 4, Acoustic file S3).

### New Caledonia 2009 Song Description

**Figure 6:**
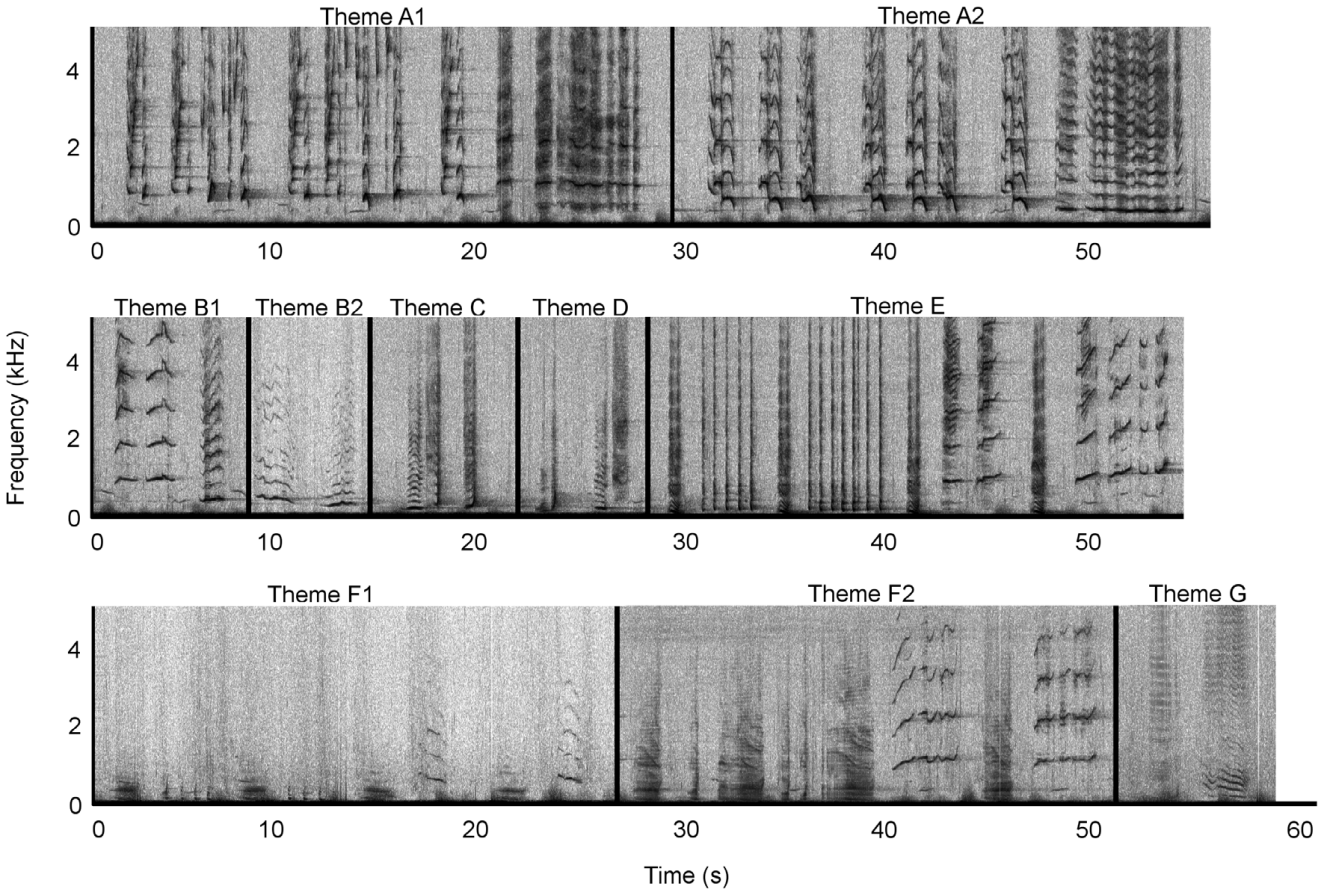
New Caledonia 2009 song. A single representative phrase is shown for each theme representing a song. Some themes contained 1 and 2 phrase types representing small but stereotyped variations to a theme. Spectrograms were generated in Raven Pro 1.4 (Blackman-Harris window, 2048 FFT size, 75% overlap). Recordings were taken from July to August 2009. Audio is provided in acoustic file S5. Note difference in frequency scale to Figure 2.

Seven themes were present in 2009, some of which contained two phrase types (Figure 6, Acoustic file S5). Themes were typically sung in the order A1, A2, B1, B2, C, D, E, F1 and F2, to form a song. Theme G, the surfacing theme, was inserted after theme D or F2 if the animal surfaced to breathe during the song. Interestingly, the first recording from New Caledonia in 2010 (July 15, 2010) contained this song type; however all background singers in the recording, and all additional singers recorded in 2010, were singing the eastern Australian song type as described above. The sequence for this particular 2010 New Caledonian singer, assessed from two songs, was B1, B2, C, D, E, and F2. This song type qualitatively matches Garland et al.’s Light Green song type [6], [51], [55].

### Theme Matching

Four of the available eight themes (H, J, L and M1) matched between the eastern Australia 2009 and 2010 song (Figures 3 and 5). All six themes from the New Caledonian 2010 song (H, I, J, K, L, M1) matched the six themes present in the eastern Australian 2009 song (Figures 3 and 4), and four of the eight possible themes (H, J, L and M1) matched the eastern Australian 2010 song (Figures 4 and 5). The Antarctic sample potentially shared five (out of a possible six) themes with the New Caledonian 2010 and eastern Australian 2009 song (H, I, J, K and M1; Figures 2–4), and three (out of a possible eight) themes with the eastern Australian 2010 song (H, J and M1; Figures 2 and 5). None of the New Caledonian 2009 themes matched the 2009 and 2010 eastern Australian, 2010 New Caledonian or 2010 Antarctic Area V themes.

## Discussion

Here we have presented the first example to our knowledge, of humpback whale song recorded on the Antarctic feeding grounds in the waters of eastern Antarctica. Themes identified from the Antarctic Area V recordings matched song themes from the two closest breeding populations which are likely to feed in this region, eastern Australia and New Caledonia. While feeding grounds along the coasts of North America (Pacific and Atlantic) are more accessible for song recording, the Southern Ocean (particularly away from the Western Antarctic Peninsula) is significantly more challenging as a study area, and this is why there is no previous record of humpback whale singing activity from this region. The opportunistic song recording documented here, and the high density of whales present near the Balleny Islands [48], has highlighted a location where future effort can be concentrated to maximize recordings of feeding ground song.

Payne and Guinee [7] hypothesized three possible mechanisms to allow acoustic contact and subsequent song transmission among populations in any ocean basin: movement of individuals from one breeding population to another between seasons, within-season movement of individuals between two breeding populations, and song exchange on shared migration routes and/or on summer feeding grounds in high latitudes. The similarity of four Antarctic Area V themes to song from the previous year from eastern Australia, and the same year (but following breeding season) in New Caledonia (confirmed by naïve observers and unlikely to be simply by chance), indicates that singing in summer on shared feeding grounds is one possible mechanism through which song can be transmitted between these populations. In addition, a recent photo-identification recapture study has indicated a low level of interchange between eastern Australia and New Caledonia (four out of 1402 individuals) [21], and these populations also share a migration route through New Zealand [56]. Thus, both between-season movement and song exchange on shared migration routes present additional possible mechanisms of song transmission within this region. Interestingly, the eastern Australian population is the largest in the region [39], [41], so if similar proportions of each population emigrate to the other in a season, this may explain its undue influence on song within the region [6] as more individuals would be expected to emigrate. While distances between these populations are small compared to populations within the North Pacific (∼1,500 km vs. ∼4,200 km), song sharing occurs with a one year delay in transmission between eastern Australia and New Caledonia [6], [51], leading to within-season differences in song despite being geographically closer than populations within the North Pacific. At this stage, however, we cannot discern among the mechanisms behind the dynamic nature of song transmission in the western South Pacific region (*i.e.,* between-season movement, shared migration routes and summer feeding grounds); we merely present that song relevant to the surrounding populations is being produced on the summer feeding grounds, the pattern of song exchange indicates song was first present in eastern Australia before being recorded in Antarctica and then New Caledonia, and that this is the first time song has been recorded in the waters of eastern Antarctica.

The sample sizes included in this study are relatively small. The sample from New Caledonia in 2009 contained three singers only (Table 2), suggesting the potential for additional variability not captured by this sample. However, the occurrence of the New Caledonian 2009 song in the first recording of the 2010 season in New Caledonia strongly suggests that, based on similar song change events in this population [6], [50], [51], it is unlikely the eastern Australian 2009 song was present and simply not recorded in New Caledonia in 2009. The data presented from Antarctic Area V are also a limited sample, but are highly suggestive due to the number of matched themes. The Balleny Islands have historically been connected through Discovery marking with eastern Australia, New Zealand and Norfolk Island (in the 1950s and early 1960s) [32], [43]. These locations have additional connections, shown through Discovery marking, photo-identification recaptures, genetic matches and satellite tagging studies with the eastern Australian and New Caledonian populations [32], [43], [46], [56], [45], [57]. Interestingly, a Discovery mark connection was made between Tonga (1958) and Area V near the Balleny Islands (1957) [42]. Song from Tonga and New Caledonia was typically the same each breeding season over the last decade, indicating a strong acoustic connection between these populations [6], [51], [55].

The 2009 New Caledonian song type was clearly different to the 2009 and 2010 eastern Australian, 2010 New Caledonian and 2010 Antarctic Area V song type. The New Caledonian 2010 song shared all six themes with the eastern Australian 2009 song. The Antarctic sample included theme I (which evolved into theme N in eastern Australia in 2010), and theme K (which was dropped from the eastern Australian 2010 song), indicating it was more related to the 2009 eastern Australian/2010 New Caledonian song. Due to the time lag in song matching between eastern Australia and New Caledonia [6], [50]–[51] (*i.e.,* the song type does not match within a season), it is likely that song was first learnt by the eastern Australian population and then transmitted at a later point in time to the New Caledonian population (through the movement of animals from eastern Australia to New Caledonia, and/or song sharing on migration, and/or in Antarctica), at which point the two populations’ songs diverged. This could occur through the divergence of migratory streams, or the movement of a group of animals away from a shared feeding aggregation (*e.g.,* around the Balleny Islands). Of particular note is the photo-identification recapture between New Caledonia (taken in 2007) and the Balleny Islands, taken on 1^st^ March 2010 as part of the same expedition [46]. This individual was genetically and behaviorally identified as a mature male; it is probable that this male was exposed to the (2009 eastern Australian) song while within the vicinity of the Balleny Islands. Although we cannot comment further as to whether this individual acquired the 2009 eastern Australian song at this specific point in time, the nearly simultaneous presence of this song with a male linked to New Caledonia clearly illustrates the potential for cultural exchange and provides direct evidence of singing while on shared summer feeding grounds in the Southern Ocean Antarctic Area V.

The present study suggests a plausible mechanism of song transmission that has major implications for our understanding of song similarity between seemingly geographically separated populations. The movement of individuals (facilitating song exchange), coupled with song transmission on the feeding grounds, may permit a rapid transfer of song across ocean basins and the circumpolar feeding grounds. Song has been introduced into the eastern Australian population from the western Australian population [13], representing a movement between two discrete breeding populations (IWC stocks D and E). This was suggested to occur through the movement of a few individuals into the population [13] as occasional movement of individuals between breeding Groups D and E has been noted [32]. Within the South Pacific, individuals have been documented feeding in Antarctic Area I where they have not traditionally been thought to feed. One male marked with a Discovery tag in Tonga (1952) was later killed in Area I (1957) [58], and two individuals (one male and one female) recently photo-identified in American Samoa were later recaptured in Area I [59]. This could facilitate the transfer of song from the western and central South Pacific region to another ‘stock’ if the males concerned sung while on the Area I feeding grounds. Song has recently been recorded on the Western Antarctic Peninsula (Area I) [26] which provides an interesting opportunity to further investigate song transmission between breeding and feeding grounds across this ocean basin.

At the broader scale it is plausible that the movement of individuals, or song transmission on the circumpolar distribution of feeding grounds in the Southern Ocean, may allow the movement of different song types from one area to the next. The movement of different versions (song types) of this acoustic sexual display is a clear example of large-scale population-wide horizontal cultural transmission in a non-human animal [6]. For the future, only through a large, international collaboration of researchers that possess song recordings across the multiple breeding (and now feeding) locations throughout the Southern Hemisphere can we investigate the intriguing possibility for a song type to undergo a complete circumpolar transmission. Such research would have significant implications for our understanding of population connectivity within the Southern Hemisphere, and would also contribute to the wider understanding of the underlying drivers for population-wide song conformity and cultural traditions in animals.

## Supporting Information

Acoustic File S1 **Antarctic Area V 2010 song.** A single representative phrase is provided for each theme and is the corresponding audio file for Figure 2.
(WAV)

Acoustic File S2 **Eastern Australia 2009 song.** A single representative phrase is provided for each theme and is the corresponding audio file for Figure 3.
(WAV)

Acoustic File S3 **New Caledonia 2010 song.** A single representative phrase is provided for each theme and is the corresponding audio file for Figure 4.
(WAV)

Acoustic File S4 **Eastern Australia 2010 song.** A single representative phrase is provided for each theme and is the corresponding audio file for Figure 5.
(WAV)

Acoustic File S5 **New Caledonia 2009 song.** A single representative phrase is provided for each theme and is the corresponding audio file for Figure 6.
(WAV)

## References

[1] Payne R, McVay S (1971) Songs of humpback whales. Science 173: 585–597 10.1126/science.173.3997.585 17833100

[2] Payne K, Payne R (1985) Large Scale Changes over 19 Years in Song of Humpback Whales in Bermuda. Z Tierpsychol 68: 89–114 10.1111/j.1439-0310.1985.tb00118.x

[3] Winn HE, Winn LK (1978) The song of the humpback whale *Megaptera novaeangliae* in the West Indies. Mar Biol 47: 97–114.

[4] Payne R (1978) Behavior and vocalization of humpback whales (Megaptera sp.). In: U.S. Department of Commerce, NTIS PB Norris KS, Reeves RR, editors. Report on a Workshop on Problems Related to Humpback Whales (*Megaptera novaeangliae*) in Hawaii. 280–794: 56–78.

[5] Payne K, Tyack P, Payne R (1983) Progressive changes in the songs of humpback whales (*Megaptera novaeangliae*): A detailed analysis of two seasons in Hawaii. In: Payne R, editor. Communication and Behavior of Whales. Boulder: Westview Press Inc. 9–57.

[6] Garland EC, Goldizen AW, Rekdahl ML, Constantine R, Garrigue C, et al (2011) Dynamic horizontal cultural transmission of humpback whale song at the ocean basin scale. Curr Biol 21: 687–691 10.1016/j.cub.2011.03.019 21497089

[7] Payne R, Guinee LN (1983) Humpbacks Whale (*Megaptera novaeangliae*) Songs as an Indicator of “Stocks”. In: Payne R, editor. Communication and Behavior of Whales. Boulder: Westview Press Inc. 333–358.

[8] Helweg DA, Herman LM, Yamamoto S, Forestell PH (1990) Comparison of Songs of Humpback Whales (*Megaptera novaeangliae*) Recorded in Japan, Hawaii, and Mexico During the Winter of 1989. Scientific Reports of Cetacean Research 1: 1–20.

[9] Helweg DA, Frankel AS, Mobley JR Jr, Herman LM (1992) Humpback whale song: our current understanding. In: Thomas JA, Kastelein RA, Supin AY, editors. Marine Mammal Sensory Systems. New York: Plenum Press. 459–483.

[10] Helweg DA, Cato DH, Jenkins PF, Garrigue C, McCauley RD (1998) Geographic variation in South Pacific humpback whale songs. Behaviour 135: 1–27.

[11] Cerchio S, Jacobsen JK, Norris TF (2001) Temporal and geographical variation in songs of humpback whales, *Megaptera novaeangliae*: synchronous change in Hawaiian and Mexican breeding assemblages. Anim Behav 62: 313–329 10.1006/anbe.2001.1747

[12] Darling JD, Sousa-Lima RS (2005) Songs indicate interaction between Humpback whale (*Megaptera novaeangliae*) populations in the western and eastern South Atlantic Ocean. Mar Mamm Sci 21: 557–566 10.1111/j.1748-7692.2005.tb01249.x

[13] Noad MJ, Cato DH, Bryden MM, Jenner M-N, Jenner KCS (2000) Cultural revolution in whale songs. Nature 408: 537 10.1038/35046199 11117730

[14] Murray A, Cerchio S, McCauley R, Jenner CS, Razafindrakoto Y, et al (2012) Song comparison reveals limited exchange between humpback whales (*Megaptera novaeangliae*) in the southern Indian Ocean. Mar Mamm Sci 28: E41–E57 10.1111/j.1748-7692.2011.00484.x

[15] Winn HE, Thompson TJ, Cummings WC, Hain J, Hudnall J, et al (1981) Song of the Humpback Whale – Population Comparisons. Behav Ecol Sociobiol 8: 41–46.

[16] Cato DH (1991) Songs of Humpback Whales: the Australian perspective. Mem Queensl Mus 30: 277–290.

[17] Razafindrakoto Y, Cerchio S, Collins T, Rosenbaum H, Ngouessono S (2009) Similarity of humpback whale song from Madagascar and Gabon indicates significant contact between South Atlantic and southwest Indian Ocean populations. Int Whal Comm: SC61/SH8.

[18] Calambokidis J, Steiger GH, Straley JM, Herman LM, Cerchio S, et al (2001) Movements and population structure of humpback whales in the North Pacific. Mar Mamm Sci 17: 769–794 10.1111/j.1748-7692.2001.tb01298.x

[19] Garrigue C, Aguayo A, Amante-Helweg VLU, Baker CS, Caballero S, et al (2002) Movements of humpback whales in Oceania, South Pacific. J Cetacean Res Manag 4: 255–260.

[20] Garrigue C, Constantine R, Poole M, Hauser N, Clapham P, et al (2011) Movement of individual humpback whales between wintering grounds of Oceania (South Pacific), 1999 to 2004. J Cetacean Res Manag (Special Issue) 3: 275–281.

[21] Garrigue C, Franklin T, Constantine R, Russell K, Burns D, et al (2011) First assessment of interchange of humpback whales between Oceania and the east coast of Australia. J Cetacean Res Manag (Special Issue) 3: 269–274.

[22] Mattila DK, Guinee LN, Mayo CA (1987) Humpback whale songs on a North Atlantic feeding ground. J Mammal 68: 880–883 10.2307/1381574

[23] Clark CW, Clapham PJ (2004) Acoustic monitoring on a humpback whale (*Megaptera novaeangliae*) feeding ground shows continual singing into late spring. Proc R Soc Lond B Biol Sci 271: 1051–1057 10.1098/rspb.2004.269914712954 PMC1691688 15293859

[24] Vu ET, Risch D, Clark CW, Gaylord S, Hatch LT, et al (2012) Humpback whale song occurs extensively on feeding grounds in the western North Atlantic Ocean. Aquat Biol 14: 175–183 10.3354/ab00390

[25] McSweeney DJ, Chu KC, Dolphin WF, Guinee LN (1989) North Pacific humpback whale songs: A comparison on southeast Alaskan feeding ground songs with Hawaiian wintering ground songs. Mar Mamm Sci 5: 139–148.

[26] Stimpert AK, Peavey LP, Nowacek DP, Friedlaender AS (2012) Humpback whale song and foraging behavior on an Antarctic feeding ground. PLoS ONE 7: e51214 10.1371/journal.pone.0051214 23284666 PMC3526533

[27] Noad MJ, Cato DH (2007) Swimming speeds of singing and non-singing humpback whales during migration. Mar Mamm Sci 23: 481–495 10.1111/j.1748-7692.2007.02414.x

[28] Norris TF, McDonald M, Barlow J (1999) Acoustic detections of singing humpbacks whales (*Megaptera novaeangliae*) in the eastern North Pacific during their northbound migration. J Acoust Soc Am 106: 506–514 10.1121/1.427071 10420640

[29] Clapham PJ, Mattila DK (1990) Humpback whale songs as indicators of migration routes. Mar Mamm Sci 6: 155–160 10.1111/j.1748-7692.1990.tb00238.x

[30] Charif RA, Clapham PJ, Clark CW (2001) Acoustic detections of singing humpback whales in deep waters off the British Isles. Mar Mamm Sci 17: 751–768 10.1111/j.1748-7692.2001.tb01297.x

[31] Kibblewhite AC, Denham RN, Barnes DJ (1967) Unusual Low-Frequency Signals Observed in New Zealand Waters. J Acoust Soc Am 41: 644–655.

[32] Chittleborough RG (1965) Dynamics of two populations of the humpback whale, *Megaptera novaeangliae* (Borowski). Aust J Mar Fresh Res 16: 33–128 10.1071/MF9650033

[33] Baker CS, Herman LM, Perry A, Lawton WS, Straley JM, et al (1986) Migratory movement and population structure of humpback whales (*Megaptera novaeangliae*) in the central and eastern North Pacific. Mar Ecol Prog Ser 31: 105–119.

[34] Baker CS, Palumbi SR, Lambertsen RH, Weinrich MT, Calambokidis J, et al (1990) Influence of seasonal migration on geographic distribution of mitochondrial DNA haplotypes in humpback whales. Nature 344: 238–240 10.1038/344238a0 1969116

[35] Clapham PJ (1996) The Social and Reproductive Biology of Humpback Whales: an Ecological Perspective. Mamm Rev 26: 27–49.

[36] International Whaling Commission (IWC) (2006) Report of the Workshop on the Comprehensive Assessment of Southern Hemisphere Humpback Whales. Int Whal Comm: SC58/Rep5.

[37] Clapham P, Mikhalev Y, Franklin W, Paton D, Baker CS, et al (2009) Catches of Humpback Whales, *Megaptera novaeangliae*, by the Soviet Union and Other Nations in the Southern Ocean, 1947–1973. Mar Fish Rev 71: 39–43.

[38] Paterson RA, Paterson P, Cato DH (2004) Continued increase in east Australian humpback whales in 2001, 2002. Mem Queensl Mus 49: 712.

[39] Noad MJ, Dunlop RA, Paton D, Cato DH (2011) Absolute and relative abundance estimates of Australian east coast humpback whales (*Megaptera novaeangliae*). J Cetacean Res Manag (Special Issue) 3: 243–52.

[40] Garrigue C, Dodemont R, Steel D, Baker CS (2004) Organismal and ‘gametic’ capture-recapture using microsatellite genotyping confirm low abundance and reproductive autonomy of humpback whales on the wintering grounds of New Caledonia. Mar Ecol Prog Ser 274: 251–262 10.3354/meps274251

[41] Constantine R, Jackson JA, Steel D, Baker CS, Brooks L, et al (2012) Abundance of humpback whales in Oceania using photo-identification and microsatellite genotyping. Mar Ecol Prog Ser 453: 249–261 10.3354/meps09613

[42] Dawbin WH (1959) New Zealand and South Pacific Whale Marking and Recoveries to the End of 1958. Norsk Hvalfangst-Tidende 48: 213–238.

[43] Dawbin WH (1964) Movements of Humpback Whales Marked in the South West Pacific Ocean 1952 to 1962. Norsk Hvalfangst-Tidende 53: 68–78.

[44] Dawbin WH (1966) The Seasonal Migratory Cycle of Humpback Whales. In: Norris KS, editor. Whales, Dolphins, and Porpoises. Berkley: University of California Press. 145–170.

[45] Steel D, Schmitt N, Anderson M, Burns D, Childerhouse S, et al. (2011) Initial genotype matching of humpback whales from the 2010 Australia/New Zealand Antarctic Whale Expedition (Area V) to Australia and the South Pacific. Int Whal Comm: SC63/SH10.

[46] Constantine R, Allen J, Beeman P, Burns D, Charrassin J-B, et al. (2011) Comprehensive photo-identification matching of Antarctic Area V humpback whales. Int Whal Comm: SC63/SH16.

[47] Gales N, Double MC, Robinson S, Jenner C, Jenner M, et al. (2009) Satellite tracking of southbound East Australian humpback whales (*Megaptera novaeangliae*): challenging the feast or famine model for migrating whales. Int Whal Comm: SC61/SH17.

[48] Gales N (2010) Antarctic Whale Expedition-Preliminary science field report and summary, *R.V. Tangaroa* Feb/Mar 2010. Int Whal Comm: SC62/O12.

[49] Garrigue C, Peltier H, Ridoux V, Franklin T, Charrassin JB (2010) CETA: a new cetacean observation program in East Antarctica. Int Whal Comm: SC62/SH3.

[50] Garland EC, Lilley MS, Goldizen AW, Rekdahl ML, Garrigue C, et al (2012) Improved versions of the Levenshtein distance method for comparing sequence information in animals’ vocalisations: tests using humpback whale song. Behaviour 149: 1413–41 10.1163/1568539X-00003032

[51] Garland EC, Noad MJ, Goldizen AW, Lilley MS, Rekdahl ML, et al (2013) Quantifying humpback whale song sequences to understand the dynamics of song exchange at the ocean basin scale. J Acoust Soc Am 133: 560–569 10.1121/1.4770232 23297927

[52] Gedamke J, Robinson SM (2010) Acoustic survey for marine mammal occurrence and distribution off East Antarctica (30–80°E) in January-February 2006. Deep Sea Res Part 2 Top Stud Oceanogr 57: 968–981 10.1016/j.dsr2.2008.10.042

[53] Mellinger DK (2001) Ishmael 1.0 User’s Guide. U.S. Department of Commerce, NOAA Technical Memo OAR-PMEL 120: 1–30.

[54] Dunlop RA, Cato DH, Noad MJ (2010) Your attention please: increasing ambient noise levels elicits a change in communication behaviour in humpback whales (*Megaptera novaeangliae*). Proc R Soc Lond B Biol Sci 277: 2521–2529 10.1098/rspb.2009.2319 PMC2894914 20392731

[55] Garland EC (2011) Cultural transmission of humpback whale song and metapopulation structure in the South Pacific Ocean. PhD thesis: University of Queensland. 208 p.

[56] Constantine R, Russell K, Gibbs N, Childerhouse S, Baker CS (2007) Photo-identification of humpback whales (*Megaptera novaeangliae*) in New Zealand waters and their migratory connections to breeding grounds of Oceania. Mar Mamm Sci 23: 715–720 10.1111/j.1748-7692.2007.00124.x

[57] Garrigue C, Zerbini AN, Geyer Y, Heide-Jørgensen M-P, Hanaoka W, et al (2010) Movements of satellite-monitored humpback whales from New Caledonia. J Mammal 91: 109–115 10.1644/09-MAMM-A-033R.1

[58] Brown SG (1957) Whale marks recovered during the Antarctic whaling season 1956/57. Norsk Hvalfangst-Tidende 10: 555–559.

[59] Robbins J, Dalla Rosa L, Allen JM, Mattila DK, Secchi ER, et al (2011) Return movement of a humpback whale between the Antarctic Peninsula and American Samoa: a seasonal migration record. Endanger Species Res 13: 117–121 10.3354/esr00328

